# The evolution of body size under environmental gradients in ectotherms: why should Bergmann's rule apply to lizards?

**DOI:** 10.1186/1471-2148-8-68

**Published:** 2008-02-27

**Authors:** Daniel Pincheira-Donoso, David J Hodgson, Tom Tregenza

**Affiliations:** 1 Centre for Ecology and Conservation, School of Biosciences, University of Exeter, Cornwall Campus, Penryn TR10 9EZ, Cornwall, UK

## Abstract

**Background:**

The impact of environmental gradients on the evolution of life history traits is a central issue in macroecology and evolutionary biology. A number of hypotheses have been formulated to explain factors shaping patterns of variation in animal mass. One such example is Bergmann's rule, which predicts that body size will be positively correlated with latitude and elevation, and hence, with decreasing environmental temperatures. A generally accepted explanation for this phenotypic response is that as body mass increases, body surface area gets proportionally smaller, which contributes to reduced rates of heat-loss. Phylogenetic and non-phylogenetic evidence reveals that endotherms follow Bergmann's rule. In contrast, while previous non-phylogenetic studies supported this prediction in up to 75% of ectotherms, recent phylogenetic comparative analyses suggest that its validity for these organisms is controversial and less understood. Moreover, little attention has been paid to why some ectotherms conform to this rule, while others do not. Here, we investigate Bergmann's rule in the six main clades forming the *Liolaemus *genus, one of the largest and most environmentally diverse genera of terrestrial vertebrates. A recent study conducted on some species belonging to four of these six clades concluded that *Liolaemus *species follow Bergmann's rule, representing the only known phylogenetic support for this model in lizards. However, a later reassessment of this evidence, performed on one of the four analysed clades, produced contrasting conclusions.

**Results:**

Our results fail to support Bergmann's rule in *Liolaemus *lizards. Non-phylogenetic and phylogenetic analyses showed that none of the studied clades experience increasing body size with increasing latitude and elevation.

**Conclusion:**

Most physiological and behavioural processes in ectotherms depend directly upon their body temperature. In cold environments, adaptations to gain heat rapidly are under strong positive selection to allow optimal feeding, mating and predator avoidance. Therefore, evolution of larger body size in colder environments appears to be a disadvantageous thermoregulatory strategy. The repeated lack of support for Bergmann's rule in ectotherms suggests that this model should be recognized as a valid rule exclusively for endotherms.

## Background

Geographical variation in environmental conditions is a major ecological factor involved in evolutionary diversification [1,2]. Since thermal regimes are particularly sensitive to latitude and altitude, geographical location imposes profound selection on organisms' metabolism, morphology and behaviour [3-5], leading to covariation between phenotypic traits and geographical gradients [5-7]. Body size is known to exhibit substantial variation in relation to thermal differences among habitats [4,8,9]. However, factors other than environmental temperature (e.g. sexual selection, predation) may also impose selection on body size [2,10-16]. As a result, models predicting patterns of evolutionary change in body mass in response to thermal variation are controversial [17-20]. One such example is Bergmann's rule [18], which suggests that species body size increases with increasing latitude and elevation, and hence, with decreasing environmental temperatures [6,21-23].

Different hypotheses have attempted to elucidate the causal factors promoting the pattern predicted by Bergmann's rule [18,24-26]. Potential explanations have focused on heat-conservation strategies [4,6,20,27], later maturation to larger body size [28,29], phylogenetic constraints [4,30], interspecific variation in migration [4], differential resistance to starvation [31-33], and effects on somatic cell sizes [18,26,34,35]. So far, the heat-conservation hypothesis, based on the fact that increases in body volume lead to decreases in relative surface area appears to be the most likely explanation [27].

Since the formulation of Bergmann's rule more than 150 years ago [21], several studies have explored the universality of its predictions across different lineages. In general, the validity of this rule appears to depend on the thermoregulatory physiology of the studied model systems. For endotherms, most evidence has supported Bergmann's rule [36-38]. This is presumably because the benefits of reducing heat-loss rates through larger body size are advantageous for cold climate organisms that maintain optimal body temperatures by metabolic generation of heat. However, the relevance of Bergmann's rule for ectotherms is less obvious, and supportive evidence is elusive [6,20,31,39].

### Bergmann's rule in ectotherms

More than 99% of species are ectotherms [40]. Consequently, no biological prediction can be considered universal if it is not supported by these organisms. The first tests to evaluate Bergmann's rule in ectotherms [41,42] claimed that up to 75% of studied species support its predictions. However, these studies were based on simplistic statistical approaches, when the importance of conducting phylogenetic comparative studies was not yet appreciated [6,31,43,44]. Unsurprisingly, the development of phylogenetic comparative analyses to test Bergmann's rule over the last decade has provided divergent lines of evidence. While a series of studies confirmed previous findings [26,27,31,39,45], others were more equivocal [18,24,26,29-31]. For example, Ray [42] gathered data from previously published studies to evaluate Bergmann's rule. Regarding fishes, this author concluded that it is "obeyed by a great number of fishes as shown by numerous reports in the literature". Curiously, no citations were provided to support this claim [29,42]. More recently however, in a rigorous study conducted on more than 600 fish populations of different freshwater species from North America, Belk and Houston [29] observed that these ectotherms tend to reverse Bergmann's rule.

With regard to tetrapod ectotherms (i.e. amphibians and reptiles), evidence is similarly controversial [31]. A number of phylogenetic studies have shown that groups of primarily aquatic and tropical-subtropical species follow Bergmann's rule. For example, some anurans, and most urodeles (salamanders and newts) and turtles exhibit a negative relationship between body mass and environmental temperatures [30,31,41]. On the other hand, debate has progressively intensified in relation to squamate reptiles (lizards, snakes). Non-phylogenetic [41] and phylogenetic [30,31] studies have revealed that squamates exhibit only a weak tendency to conform to this model. Nevertheless, research conducted on some widespread groups has concluded that these reptiles can exhibit body size trends predicted by Bergmann's rule [20,27,46]. For example, a recent phylogenetic study conducted on *Liolaemus *lizards supported this temperature-size model [27]. Cruz *et al. *[27] observed that species of the clade *Liolaemus boulengeri *[11] show larger body size at higher latitudes and elevations. An additional integrative analysis including species belonging to congeneric clades led Cruz *et al. *[27] to conclude that "the strong, positive, size-latitude relationship in the *L. boulengeri *clade apparently accounted for the pattern observed for the entire dataset". However, after enlarging the sample of taxa belonging to one of the studied clades (clade *boulengeri*), Pincheira-Donoso *et al. *[39] observed that these species do not support Bergmann's rule.

Among factors that might explain the disparity observed across studies testing Bergmann's rule in ectotherms, the limited availability of phylogenetic studies is perhaps the most obvious [29,31,47]. Only scarce or generalized tests have been conducted on ectotherms, and most of this research focuses on the analyses of few species per clade (e.g. a single species representing clades consisting of more than 100 taxa) [20,31]. Moreover, studies focused on analysing explicit patterns of body size variation in the species of the same clade in response to continuous geographical gradients are still rare [27,29,46]. Recent criticism suggests that a rigorous test of Bergmann's rule should focus on species belonging to a monophyletic clade exhibiting substantial variation in patterns of body size and occurring in a broad geographical area encompassing a wide range of environmental conditions [6,39,46].

Lizards of the South American genus *Liolaemus *offer unique opportunities to test predictions concerning the impact of selection on traits recognized as labile to variation in environmental temperatures, such as body size [11,27,48]. This clade represents one of the most diverse known amniote lineages. Consisting of more than 190 named species, *Liolaemus *occurs in the widest range of environmental conditions recorded for any lizard genus [49-53]. These iguanians range from tropical-subtropical areas in Brazil and Peru, and the Atacama Desert in Chile, to austral Patagonia in Tierra del Fuego, the southernmost place where reptiles have been recorded [53-59]. The altitudinal distribution of *Liolaemus *is also one of the broadest known among squamate reptiles, occurring from sea level to over 5000 m in the Andes range [48,50,57,60,61]. These biological features satisfy all of the requirements recognized as essential for a model group employed to test predictions concerning evolutionary radiations [6,39,46].

**Figure 1:**
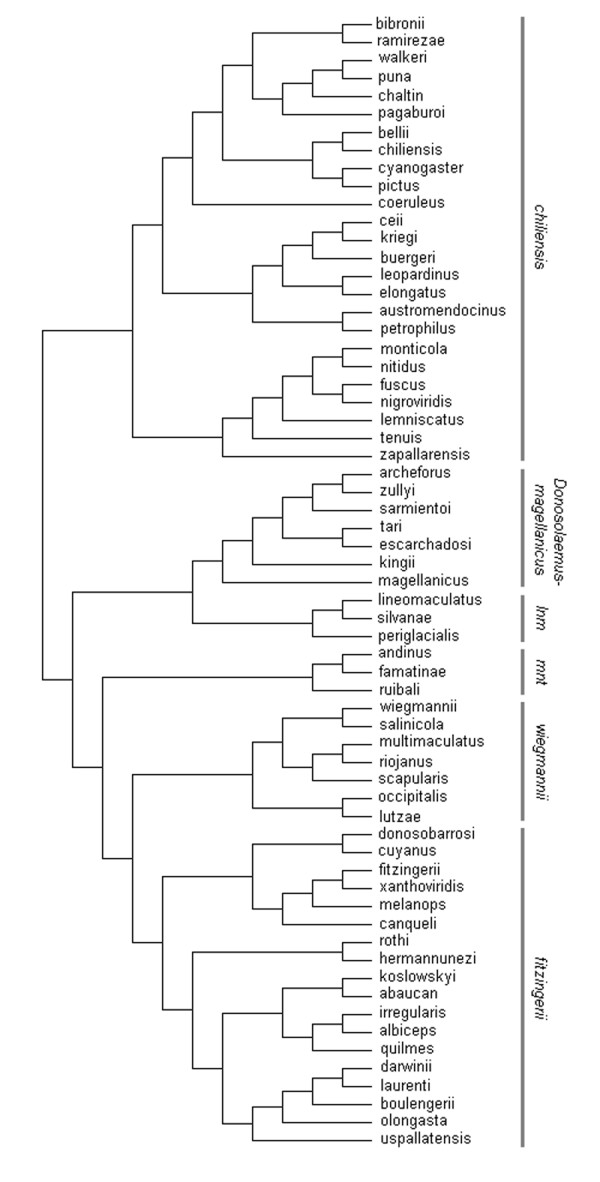
Phylogenetic relationships for 63 of the 126 *Liolaemus *taxa included in this study. The clades *lineomaculatus *(*lnm*) and *montanus *(*mnt*) are abbreviated.

Here, we investigate the effect that continuous variation in environmental temperatures imposes on the evolution of body size among species of the *Liolaemus *genus, using a comparative approach. We studied a set of more than 120 species (see additional file 1: Supplementary table), 63 of which are included in an explicit phylogenetic hypothesis (Fig. 1). This species sample represents almost the entire biogeographical, ecological and morphological diversity known for these lizards. We aim to test the hypothesis that increasing latitude and elevation (and therefore, decreasing environmental temperatures) are associated with larger body size [6,21]. We suggest as an alternative hypothesis that large body size is disadvantageous for ectotherms in cold-climates, because it demands longer time basking to achieve optimal metabolic temperatures. Therefore, we expect to find weak or no evidence in support of Bergmann's rule.

## Results

### Body size patterns under environmental gradients

We found non significant relationships between environmental gradients and body size in most of the tests performed. In none of the studied clades was Bergmann's rule supported (Table 1). For both methods of data analysis, significantly high or low standardized residual (*z*-scores) values revealed the presence of outliers which might affect the accuracy of the models. For independent clade analyses, extreme *z*-scores (see methods) were found in the clades *montanus *(one case with *z*-scores > 2.05) and *Donosolaemus-magellanicus *(one case with *z*-scores < -2.26). The relationship between independent contrasts for body size and adjusted latitudinal midpoint (ALM, latitude adjusted for altitude, see below) revealed the presence of five outliers affecting the model (over 8.06% of the cases with *z*-scores > 2.12, and < -2.21). The observed differences in the correlation and regression analyses including and excluding outliers are detailed below.

**Table 1:** Results of least squares regression analyses conducted on clades separately (non-phylogenetic) and on independent contrasts (phylogenetic) in the genus *Liolaemus*. In the clades *Donosolaemus-magellanicus *and *montanus*, these analyses were conducted including outliers (IO) and excluding outliers (EO). See methods for details.

Clade	N	*R*^2^	*r*	*F*	*df*	*P*
**Non-Phylogenetic**						
*chiliensis*	56	0.009	0.09	0.471	1,54	0.495
*Donosolaemus-magellanicus *(IO)	12	0.009	-0.09	0.086	1,10	0.775
*Donosolaemus-magellanicus *(EO)	11	0.088	0.29	0.869	1,9	0.375
*fitzingerii*	22	0.023	0.15	0.467	1,20	0.502
*lineomaculatus*	4	0.3	0.55	0.855	1,2	0.453
*montanus *(IO)	21	0.165	-0.41	3.75	1,19	0.068
*montanus *(EO)	20	0.313	-0.56	8.191	1,18	0.01
*wiegmannii*	8	0.122	-0.35	0.837	1,6	0.396

**Phylogenetic**						
*Liolaemus *genus (IO)	63	0.023	-0.15	1.439	1,61	0.235
*Liolaemus *genus (EO)	58	0.064	-0.25	3.842	1,56	0.055

**Figure 2:**
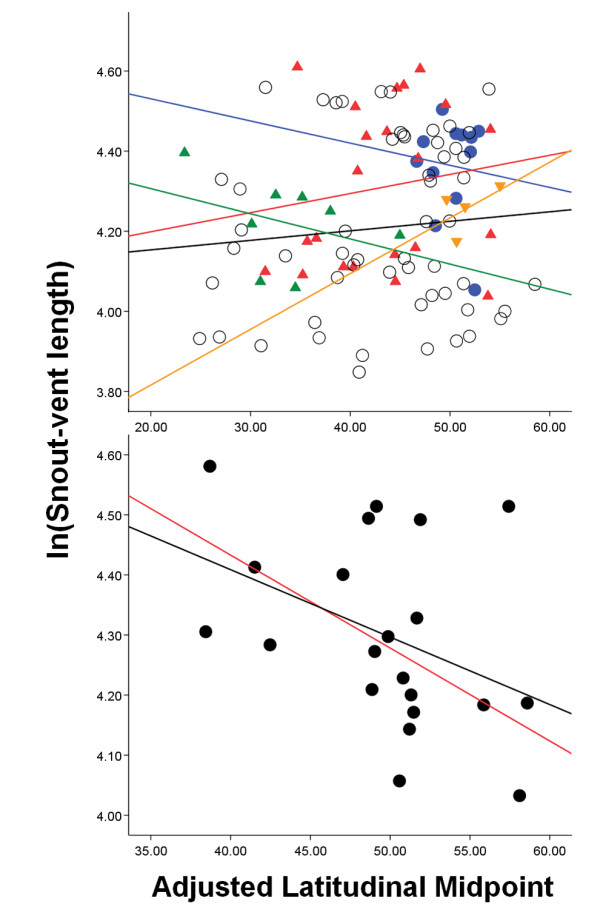
Linear regressions of raw data for ln(snout-vent length) against adjusted latitudinal midpoint in the six main clades forming the *Liolaemus *genus. Top: regressions for the clades *chiliensis *(black), *Donosolaemus-magellanicus *including outliers (blue; slope from analysis excluding outliers not shown, as it provided identical qualitative results), *fitzingerii *(red), *lineomaculatus *(orange), and *wiegmannii *(green). Bottom: regressions for the clade *montanus *including (black) and excluding (red) outliers.

Linear bivariate regression analyses showed that body size does not vary predictably with adjusted latitudinal midpoint (ALM) in the genus *Liolaemus*. For analyses conducted on clades separately, low proportions of variance were explained in the groups *chiliensis*, *fitzingerii*, *wiegmannii *and *lineomaculatus *(Table 1). Regression analyses including outliers showed non-significant relationships between SVL and ALM in the clades *montanus *and *Donosolaemus-magellanicus*. When excluding outliers, we found a significant negative relationship between SVL and ALM for the clade *montanus *and a non-significant relationship in the clade *Donosolaemus-magellanicus *(Table 1; Fig. 2).

**Figure 3:**
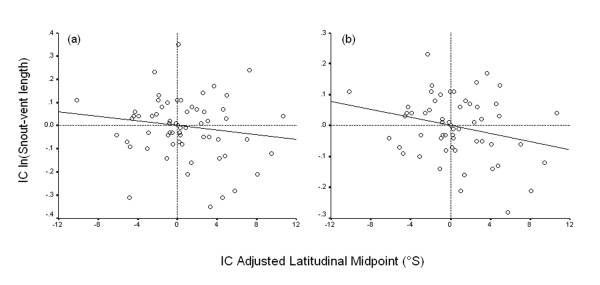
Linear regression analyses (through the origin) of phylogenetically independent contrasts (IC) for ln(snout-vent length) against adjusted latitudinal midpoint in the entire dataset of *Liolaemus *species for which phylogenetic information was available. (a) Linear regression observed when including outliers, and (b) when excluding outliers.

Linear regression analyses conducted on phylogenetic independent contrasts (through the origin) revealed that ALM cannot predict body size when including outliers (Table 1; Fig. 3a), and when excluding outliers (Table 1; Fig. 3b).

### Effects of sample size reduction

Non-phylogenetic linear regressions conducted on the 63 species for which phylogenetic information was available (see Additional file 1: Supplementary table), revealed that reduction of sample size for phylogenetic analyses (from 126 to 63 species; see methods) does not produce qualitative differences in the results. As observed in the whole dataset (126 species), body size does not vary predictably with adjusted latitudinal midpoint (ALM) in any of the studied groups. For these 63 species, non-significant proportions of variance were explained in the clades *chiliensis *(*R*^2 ^= 0.063, *F*_1,23 _= 1.55, *P *= 0.23), *Donosolaemus-magellanicus *(*R*^2 ^= 0.14, *F*_1,5 _= 0.78, *P *= 0.42), *fitzingerii *(*R*^2 ^= 0.17, *F*_1,16 _= 3.22, *P *= 0.092), *lineomaculatus *(*R*^2 ^= 0.79, *F*_1,1 _= 3.66, *P *= 0.31), *montanus *(*R*^2 ^= 0.17, *F*_1,1 _= 0.21, *P *= 0.73) and *wiegmannii *(*R*^2 ^= 0.16, *F*_1,5 _= 0.92, *P *= 0.38). The analysis on standardized residuals confirmed that none of the regression models suffered from oultiers.

## Discussion

This analysis of Bergmann's rule is unique in including a representative proportion of the geographical, ecological, morphological and phylogenetic diversity of one of the most species rich terrestrial ectotherm vertebrate genera, *Liolaemus *lizards [see [6,39,46]]. Our results fail to support Bergmann's rule. Non-phylogenetic and phylogenetic analyses showed that increasing latitude and elevation do not predict increasing body size in *Liolaemus *species. These results further contradict the only known phylogenetic evidence in favour of Bergmann's rule reported in squamate reptiles [[27], see also [39]], and a recent large-scale analysis conducted on assemblages of European lizards supporting this macroecological model [20].

### Thermal gradients, thermoregulatory physiology and body size

Research on Bergmann's rule has inspired debate about why increasing body size should be advantageous for cold climate species, and why its predictions are observed in some groups but not in others [6,31,39]. For example, it has been suggested that the anatomical characteristics of the skin (e.g. density, or epidermal covering such as feathers and fur) in groups as different as birds, mammals and turtles make large body size advantageous in colder environments and that the absence of these skin characteristics in squamate reptiles explains why they generally fail to follow Bergmann's rule [6,31,62]. In contrast, much less reference has been made to the role that thermoregulatory physiology of organisms (endothermy and ectothermy) can play during the process of body size evolution [39]. Indeed, an important part of the available discussions focuses on the benefits of increasing body size for heat retention in cold climate species. However, since conservation of body heat in ectotherms is only possible once they have optimally achieved it (which is metabolically resolved in endotherms), it appears to be simplistic to exclude the fact that increasing body mass also reduces the rates of heat gain [31], which may be as critical as the need for retaining heat [6,32]. This is illustrated, for example, by the observation that gravid females in several lizard species tend to bask more often or for longer than non-gravid females [63-66].

It might be argued that lizard body size and heating rates do not covary in a linear fashion, as these organisms may adjust (plastically or genetically) some physiological and behavioural traits to increase heating rates in cold environments [5,7,20,67,68]. For example, it has been observed that physiological adaptations may increase heating rates up to 17% in cold climate lizards [20,68]. Likewise, behavioural adjustments such as selection of basking sites less exposed to wind [3] or modifications in the postures and body orientation to the sun may contribute to gain heat more rapidly [3,69,70]. However, even though these adaptations contribute to increase heating rates in lizards inhabiting cold environments, their efficiency has limits. Indeed, a number of studies have shown that annual and daily activity, and thermoregulatory processes in cold climate lizards may vary substantially in comparison to warm climate lizards. For example, it has been observed that high elevation (e.g. over 4000 m) lizards tend to exhibit considerably shorter daily activities, often no more than four or five hours a day [71-73], than species distributed in lower latitudes and elevations, which may remain active for over twelve hours per day [57,61,72,74-76]. One of the most plausible factors to explain this pattern is that mean daily temperatures are lower, hot hours per day are fewer, and warm seasons are shorter in cold climates [22,71,77-79]. Therefore, even in presence of the above mentioned physiological and behavioural adjustments, cold environments restrict severely the patterns of activity in lizards, as a consequence of their failure to overcome the selective pressures imposed by low temperatures. This suggests that the thermoregulatory limitations determined by the ectothermal condition of lizards might be one of the main (if not the main) factors constraining the maximum attainable limits of body size in cold climate species.

Large body mass in cold climate lizards would have dramatic consequences for ecological performance (imposed by natural selection) and reproductive success (imposed by sexual selection), because in conditions of suboptimal metabolic temperatures most physiological functions (and hence, behavioural responses) occur at suboptimal rates [3,80]. For example, performance at prey capture, predator evasion, endurance, digestion, mate courtship, sperm production and conversion of lipids is substantially reduced at suboptimal body temperatures [3,66,80-83]. Also, basking for longer periods may increase the risk of predation by diurnal hunters [66]. Consequently, large body mass in cold climate lizards would be disadvantageous. Hence, the hypothesis of heat conservation cannot be accepted (at least in these organisms), as it predicts increasing body size in low-temperature environments. These claims are supported by a number of previous studies conducted in lizards [30,31,39].

Finally, it might also be argued that large ectotherms can maintain higher constant minimum body temperatures than do smaller species. However, it is unlikely that even relatively large cold climate lizards are able to retain heat overnight. Indeed, it has been observed in several species [84] that during the initial period of basking, the body temperature of lizards is very similar to the environmental temperature. Additionally, field observations conducted at high latitudes and elevations in South America (DPD, unpublished data) reveal that different sized *Liolaemus *lizards exhibit similar temperatures before initiating basking.

### Trophic niche evolution and body size

The evolution of trophic niches in lizards may provide additional evidence to support the idea that larger body size is disadvantageous for species living in cold environments. Although most lizards feed on other animals, omnivory and herbivory have evolved independently many times among these squamates [80,85]. Plant matter is a low energy food for lizards [3]. Therefore, omnivory and herbivory are advantageous for these reptiles only when certain morphological and thermoregulatory conditions are met. In general, it has been observed that plant consumption has evolved primarily in species occurring in warm habitats, where vegetation is extremely abundant (e.g. tropical forests), or where abundance of animal prey is extremely low (e.g. deserts, small islands) [11,80,86,87]. In these environments, lizards can attain large body size and high body temperatures, both traits considered essential requirements for efficient digestion of plant matter [3,88-90]. A large body allows for a more voluminous gut, and high body temperature provides optimal conditions for endosymbionts (bacteria and protozoa) specialized in breaking down otherwise indigestible plant matter [3,9,91-93]. Consequently, according to these rules of herbivory [3,11], most lizards in which plant consumption has evolved are expected to reverse Bergmann's rule, because larger species tend to occur in warm environments.

Contrary to predictions, in a recent study Espinoza *et al. *[11] found that omnivory and herbivory have not only evolved in warm-climate and large lizards, but also in small species occurring in cold environments [94]. These authors observed that a large number of small Liolaemidae species (which includes *Liolaemus*) restricted to high latitudes or elevations, have substantial proportions of plant matter in their diets (i.e. 11%–100% of digestive volume content). Although the body size pattern observed in omnivorous and herbivorous Liolaemidae species differs significantly from the pattern known for plant-consumer lizards occurring in warm climates, the explanation may be exactly the same. Since digestion of plant matter requires the highest possible body temperatures [11,93], evolution of large body size in cold-climate Liolaemidae species would decrease the thermoregulatory efficiency needed to achieve optimal metabolic temperatures. This in turn would affect negatively the digestion of plant matter. Therefore, in the case of Liolaemidae species occurring at high latitudes and elevations, evolution of large size would be disadvantageous, challenging patterns predicted by Bergmann's rule.

### Why do some ectotherms appear to follow Bergmann's rule?

Thermal environment differs from some other sources of selection in that it is a dominant selective force at low temperatures [31,39], but at higher temperatures, ectotherms are essentially freed from thermal constraints and hence other factors may become dominant in determining body size. These include fecundity selection [10,13,14], reduction of mass-specific energy requirements [3], greater physiological homeostasis [9], and reduction of mortality rates by predation [2,95-97]. Therefore, ectotherm clades whose geographical distributions are primarily restricted to tropical or subtropical ecosystems, such as continental turtles [30,31], may exhibit increasing body size with increasing latitude and moderate elevations. This pattern probably reflects a response to selective factors other than the thermal environment which is essentially benign throughout the geographic range of the group. Alternatively, observations of Bergmann's clines in some ectotherm vertebrates (i.e. turtles, salamanders) may be explained by the large taxonomic scale at which studies have been conducted [30,31] which could mean that different patterns of body size variation at an interspecific scale might be obscured by patterns observed at inter-clade scales [27]. Consequently, for example, although Ashton [45] observed that salamanders tend to follow Bergmann's rule, we could expect that a test incorporating a substantially larger number of species from a wider diversity of areas find that these amphibians do not follow Bergmann's clines (the situation might be different for turtles, as these animals are more restricted to tropical zones).

## Conclusion

Physiological processes in ectotherms are strongly dependent upon their body temperature [3,80] with numerous consequences for survival and reproduction. Since heating rates in ectotherms are critically determined by body mass, large body size in species occurring at high latitudes or elevations is likely to be disadvantageous. We found no evidence to support Bergmann's rule in *Liolaemus *lizards: increasing latitude and elevation are not associated with larger body size in these reptiles (Fig. 2). We suggest that Bergmann's rule should be recognized as a macroecological prediction employed to investigate patterns of body size evolution only in endotherms, as it was originally proposed by Bergmann [21].

## Methods

### Phylogenetic scale and study species

Even though many studies testing Bergmann's rule have focused on intraspecific comparisons [19,34,37,73,98,99], the rule [6,21,23] was originally developed on the basis of multiple species comparisons [21] which is the approach that we took. Meiri & Thomas [100] provided a detailed discussion on the taxonomical scale of Bergmann's rule in an historical context.

We gathered data on body size with respect to latitude and elevation from 4942 adult *Liolaemus *specimens of both sexes representing a total of 126 species from museum collections (see appendix and Additional file 1: Supplementary table). Since body size of lizards may be affected by distribution in islands [101-103], we excluded insular *Liolaemus *taxa from our dataset (e.g. *L. brattstroemi*, *L. melaniceps*). Collection numbers and localities for most of the studied species and specimens can be found in Pincheira-Donoso & Núñez [48]. The species forming our sample cover a representative proportion of the total biogeographical, ecological, morphological and taxonomical diversity of the genus *Liolaemus*. The studied taxa belong to the clades *chiliensis*, *Donosolaemus-magellanicus*, *fitzingerii*, *lineomaculatus*, *montanus *and *wiegmannii *[11,48,57]. These groups represent the six main lineages known for this genus. The studied species encompass almost the entire geographical range known for *Liolaemus *[57,104]. The total dataset comprises individuals coming from austral Patagonia in Argentina and Chile, the high Andes plateau (> 4500–5000 m), the Atacama Desert, tropical areas in eastern Brazil, austral forests in southern Chile and several intermediate and temperate areas [48,50,51,54,57,60,104].

### Environmental estimations

Since environmental temperatures decrease with increasing latitude and altitude [27,45,78], it is necessary to account for the combined effect of latitude and elevation when testing thermal dependence of traits [27]. Latitudinal and altitudinal data were used as estimators of the thermal conditions under which species live by converting them to a single Adjusted Latitudinal Midpoint (ALM) for each species. This scale, recently calibrated by Cruz *et al. *[27], and similar versions have been used for estimations of species' environmental conditions in comparative studies [11,39,105,106]. The ALM is obtained assuming that environmental temperature in altitudinal transects declines 0.65°C for each 100 m of increased elevation [27,78]. Cruz *et al. *[27] obtained a corrected latitudinal value for latitude and altitudinal thermal covariation using the formula *y *= 0.009*x *- 6.2627, where *x *represents the altitudinal midpoint for each species, and *y *the corrected temperature for latitude which is added to the latitudinal midpoint for each species (the details of this formula were provided personally by F.B. Cruz, as they are not published in the same format in Cruz *et al. *[27]). The final value is referred to as the adjusted latitudinal midpoint for South American areas where *Liolaemus *occurs, calculated for each species [27].

### Statistical analysis and phylogenetic control

To investigate the effects of environmental variation on *Liolaemus *species size, we used snout-vent length (SVL) as a proxy for body size. Snout-vent length is a widely used measure of body size in squamate reptiles. This parameter is positively correlated with other body variables, such as mass, and with ecological and life-history traits [3,27,86]. Several recent studies have selected the largest recorded values for SVL as an estimation of species size [27,57,107]. However, utilization of single extreme values may lead to misleading results when testing these kinds of hypotheses [108,109]. Recent evidence [110] shows that estimation of asymptotic size can be reasonably obtained using the largest individual per sample, but only in lineages that follow asymptotic growth curves, such as *Anolis *lizards [110]. Since it is unknown whether *Liolaemus *follow asymptotic growth curves [39], the use of the largest recorded individual per sample may bias analyses. An alternative method that offers greater statistical power is the estimation of body size by obtaining intermediate percentiles between the maximum record and the sample mean. For example, it has been found in agamid species that the use of mean sample values may lead to substantial underestimation of asymptotic size, whereas the use of the largest individual per sample may lead to overestimation [108]. In contrast, these intermediate percentiles (between mean and maximum record) provide the most accurate estimate of asymptotic size. Moreover, such percentiles have low variance, low dependence on sample size, and are amenable to bootstrap estimations of confidence intervals, when compared to the largest individual per sample [108]. Consequently, we used the mean SVL of the largest two-thirds of the adults for each studied species [111]. Since species of the genus *Liolaemus *exhibit various patterns of sexual size dimorphism (i.e. larger males, larger females or no sexual dimorphism) [48,50,54,57,60,61,112,113], SVL mean values were calculated for males and females, to estimate a mean value for the species [27,39]. Whenever possible (117 of 126 species) samples comprised a similar number of males and females.

Prior to analyses SVL values were ln-transformed. This ln-transformation reduces skewness of the original measurements, and helps to homogenize variance [114-116]. After ln-transformation, SVL met the statistical assumptions required for parametric analyses (according to Shapiro-Wilk tests).

It is now well appreciated that samples consisting of species sharing phylogenetic histories cannot be considered independent evolutionary entities in comparative analyses [43,117]. Consequently, simple statistical analyses might provide phylogenetically biased evidence, necessitating the development of explicit approaches to control the impact of shared ancestry [43,44,118]. On the other hand, recent studies have shown that conventional regression and correlation analyses on raw data may perform better than independent contrasts data under certain conditions of phenotypic evolution [119-122]. For example, Carvalho *et al. *[121] suggested that it is not always necessary to perform phylogenetic simulations for statistical analyses when there is little phylogenetic effect, and that comparative analyses in some cases should be applied as a conservative approach. Hence, providing results from both conventional statistical procedures and explicit phylogenetic analyses may be a more robust approach [2,119]. In the case of body size patterns observed in *Liolaemus *species, the distribution of this trait across the phylogeny may reveal only a partial effect of shared ancestry. Therefore, we analyzed our data in two different ways. First, using conventional ordinary least squares bivariate regression analyses on raw ln-transformed data, separately for each of the main clades [43,44]. We identified these clades as detailed above following the latest available nomenclature [11,27,48,57] (Fig. 1). Each of the recognized clades has previously been supported by different phylogenetic hypotheses based on molecular, morphological and combined molecular and morphological evidence [11,27,51,57,59,123].

Second, we analyzed the dataset using an explicit phylogenetic approach, by calculating independent contrasts [43] as implemented in COMPARE version 4.6b [124]. We examined species relationships using a phylogenetic hypothesis of the genus *Liolaemus *derived from different recent studies [11,27,51,59,123]. Because this phylogeny (Fig. 1) is based on both molecular and morphological data, we performed phylogenetic analyses under a speciational Brownian motion model of evolutionary change, assuming branch lengths equal to 1.0 [11,125-127]. Since phylogenetic information was only available for 63 of the 126 species included in our dataset (see additional file 1: Supplementary table), we conducted all analyses in three different ways to test for the potential effect that reduction of sample size can have on the results: (i) ordinary least squares regressions on raw data separately for each of the six clades, including the entire species sample (i.e. 126 species), (ii) the same regression analyses (on raw data) for separate clades conducted in the entire dataset, but using only the 63 species included in the phylogeny, and (iii) ordinary least squares regression analyses (through the origin) on phylogenetically independent contrast for those species for which phylogenetic information was available. Since the existence of outliers may have large effects on statistical analyses [116], we performed all the regressions including and excluding outliers. Data points with standardized residuals (i.e. *z*-scores) greater than 2 or less than -2 were considered outliers [114]. Except for one analysis (non-phylogenetic regression on the *montanus *clade), regressions including and excluding outliers did not differ qualitatively.

## Authors' contributions

DPD participated in the design of the study, conducted all the field work, performed the statistical and phylogenetic analyses and wrote the manuscript.

DJH participated in the design of the study, in the statistical analyses and in the writing of the manuscript.

TT participated in the design of the study, in the statistical analyses and in the writing of the manuscript.

## Appendix

The studied material is housed in the herpetological collections of the following institutions. Collections identified with an asterisk (*) indicate the existence of specimens with collection data, but without an official collection number at the moment of our study. Division Reptiles and Amphibians, Museo Nacional de Historia Natural de Chile (MNHN*), Zoological Museum, Facultad de Ciencias Naturales y Oceanograficas, Universidad de Concepcion, Chile (MZUC*), Division Zoology, Museo de Historia Natural de Concepcion, Chile (MHNC*), Department of Cell Biology and Genetics, Facultad de Medicina, Universidad de Chile (DBCGUCH*), Instituto de Biologia Animal, Facultad de Ciencias Agrarias, Universidad Nacional de Cuyo, Argentina (IBAUNC*), Instituto Argentino de Investigaciones de las Zonas Aridas, CRICYT, Argentina (IADIZA), Natural History Museum of London (NHML), Jose Miguel Cei Diagnostic Collection (JMC-DC), Jose Alejandro Scolaro Diagnostic Collection (JAS-DC), and in the Herpetological Collection of the senior author, D. Pincheira-Donoso (CHDPD*).

## Supplementary Material

Additional file 1 **Supplementary Table**. Snout-vent length (SVL), latitudinal and altitudinal range of the species included in this study. Taxa for which phylogenetic information is available are indicated in bold. Click here for file
