# Supplementary material for: The evolution of body size under environmental gradients in ectotherms: why should Bergmann's rule apply to lizards?
Source: BMC Evol Biol. 2008 Feb 27;8:68. doi: 10.1186/1471-2148-8-68 (PMC2268677; doi:10.1186/1471-2148-8-68)
Supplement: Additional file 1 — Supplementary Table. Snout-vent length (SVL), latitudinal and altitudinal range of the species included in this study. Taxa for which phylogenetic information is available are indicated in bold. [file 1471-2148-8-68-S1.doc]

**Table 1.** Snout-vent length (SVL), latitudinal and altitudinal range of the species included in this study. Taxa for which phylogenetic information is available are indicated in bold.

| Species | N | SVL (mm) | Latitudinal  Range (°S) | Altitudinal  Range (m) |
| --- | --- | --- | --- | --- |
| *Liolaemus*  *alticolor*  *araucaniensis*  *atacamensis*  ***austromendocinus***  *barbarae^a^*  ***bellii***  ***bibronii***  *bisignatus*  ***buergeri***  ***ceii***  cf. *elongatus*  ***chaltin***  ***chiliensis***  *chillanensis*  ***coeruleus***  *constanzae*  *cristiani*  *curicensis*  *curis*  ***cyanogaster***  ***elongatus***  *fitzgeraldi*  ***fuscus***  *gravenhorstii*  *gununakuna*  *hellmichi*  *isabelae*  ***kriegi***  ***lemniscatus***  ***leopardinus***  *lorenzmuelleri*  *maldonadae*  ***monticola***  *moradoensis*  *neuquensis*  *nigromaculatus*  ***nigroviridis***  ***nitidus***  ***pagaburoi***  *paulinae*  ***petrophilus***  ***pictus***  *platei*  *pseudolemniscatus*  ***puna****^a^*  ***ramirezae***  *ramonensis*  *schroederi*  *silvai*  *tacnae*  ***tenuis***  *thermarum*  *valdesianus*  *velosoi*  ***walkeri***  ***zapallarensis***  *Donosolaemus-magellanicus*  ***archeforus***  *baguali*  ***escarchadosi***  *gallardoi*  ***kingii***  ***sarmientoi***  *scolaroi*  *somuncurae*  ***tari***  *tristis*  ***zullyi***  ***magellanicus***  *montanus*  ***andinus***  *eleodori*  *erguetae*  *fabiani*  ***famatinae***  *foxi*  *hajeki*  *jamesi*  *multicolor*  *nigriceps*  *pantherinus*  *patriciaiturrae*  *pleopholis*  *puritamensis*  *robertoi*  *rosenmanni*  ***ruibali***  *signifer*  *stolzmanni*  *vallecurensis*  *fitzingerii*  ***abaucan***  ***albiceps***  ***boulengerii***  ***canqueli***  ***chacoensis***  ***cuyanus***  ***darwinii***  ***donosobarrosi***  *enigmaticus*  ***fitzingerii***  ***hermannunezi***  ***irregularis***  ***koslowsky***  ***laurenti***  *loboi*  *mapuche*  ***melanops***  *morenoi*  ***olongasta***  *ornatus*  ***quilmes***  ***rothi***  *sagei*  *tehuelche*  ***uspallatensis***  ***xanthoviridis***  *wiegmannii*  ***lutzae***  ***multimaculatus***  ***occipitalis***  *rabinoi*  ***riojanus***  ***salinicola***  ***scapularis***  ***wiegmannii***  *silvanae*  *kolengh*  ***lineomaculatus***  ***periglacialis***  ***silvanae*** | 32  11  26  9  19  173  26  23  17  11  14  7  138  19  36  146  6  65  55  48  27  29  74  22  6  26  19  27  467  26  23  3  133  59  26  19  411  64  14  44  8  131  41  9  8  6  16  94  12  3  462  8  21  44  8  110  13  16  4  11  24  14  34  8  17  6  18  33  47  51  2  51  10  24  27  21  23  19  26  29  14  23  32  58  331  6  4  12  10  15  18  9  11  16  31  5  2  23  11  28  12  8  8  12  11  5  11  31  9  18  6  9  44  7  11  6  13  6  12  11  11  34  14  36  17  12 | 51.3  60.9  74.1  94.5  50.7  68.4  49.7  75.9  80.2  84.4  83.9  56.8  91.9  68.3  57.1  66.7  76.2  60.2  94.4  63.1  76.7  54.8  48.9  62.7  92.2  51.0  80.3  85.3  51.1  86.7  95.1  83.2  62.3  58.4  58.5  58.6  75.6  92.6  54.6  53.1  84.8  61.3  63.9  50.1  53.6  55.5  85.3  61.1  66.9  46.9  59.4  82.0  85.8  51.2  62.1  95.5  85.1  84.8  85.6  81.3  83.4  84.3  67.6  79.4  90.4  77.2  72.4  57.6  65.8  73.5  65.6  74.1  56.4  81.5  67.3  91.3  63.0  91.3  64.8  89.5  68.6  97.6  66.7  71.7  57.8  75.8  89.3  72.5  59.8  91.5  77.5  96.0  52.8  100.5  64.0  61.0  56.7  100.0  58.8  86.0  65.5  60.3  64.7  78.4  80.0  84.5  65.0  66.1  61.0  95.3  85.5  70.1  62.9  91.0  81.1  70.1  67.9  66.0  58.8  73.0  72.6  57.9  72.2  65.0  70.9  74.4 | 17°00’-21°35’  37°28’-38°50’  23°55’-28°30’  34°30’-36°20’  22°40’-23°13’  33°11’-33°21’  32°00’-49°00’  26°20’-27°50’  36°00’-38°50’  34°55’-38°48’  34°17’-34°17’  21°53’-22°42’  31°22’-39°24’  36°50’-39°27’  38°38’-38°42’  22°37’-23°55’  35°36’-35°38’  34°08’-35°03’  35°48’-35°48’  36°40’-41°45’  29°00’-46°00’  32°46’-32°55’  30°30’-36°35’  33°25’-33°35’  37°55’-39°30’  23°28’-23°28’  26°14’-26°26’  34°00’-42°04’  30°26’-39°40’  33°15’-33°21’  29°49’-30°13’  30°43’-30°43’  33°11’-34°11’  33°42’-33°45’  37°47’-37°51’  23°50’-28°30’  32°58’-34°04’  28°15’-36°20’  26°44’-27°30’  22°27’-22°28’  41°20’-43°50’  35°27’-43°23’  25°00’-31°38’  29°56’-32°10’  21°40’-28°20’  24°20’-27°20’  33°24’-33°30’  33°16’-36°37’  29°05’-29°05’  17°37’-18°04’  32°01’-41°44’  35°15’-35°15’  33°47’-33°56’  26°23’-27°23’  11°50’-11°56’  30°00’-33°00’    46°38’-47°10’  49°10’-49°30’  50°30’-50°40’  47°33’-47°55’  43°00’-51°40’  52°00’-52°15’  46°49’-46°52’  40°44’-40°50’  49°12’-49°16’  46°50’-47°00’  46°42’-47°13’  51°00’-53°57’  22°43’-26°00’  29°06’-29°10’  22°00’-22°25’  22°55’-23°45’  28°45’-28°55’  22°41’-22°44’  21°19’-22°20’  17°00’-20°55’  21°40’-23°05’  24°00’-28°42’  16°23’-21°42’  26°14’-26°26’  18°12’-18°12’  22°55’-22°55’  29°47’-30°28’  26°27’-28°42’  32°27’-32°55’  16°35’-22°47’  21°29’-22°50’  29°34’-29°39’  27°19’-27°47’  23°30’-24°26’  34°00’-42°00’  43°00’-44°03’  22°57’-34°03’  27°19’-33°00’  28°28’-42°55’  36°30’-36°40’  18°13’-18°13’  44°00’-50°00’  37°30’-37°32’  23°55’-24°11’  27°11’-29°18’  28°10’-30°12’  41°00’-41°00’  38°33’-39°03’  36°26’-43°00’  38°47’-41°06’  28°38’-31°14’  22°00’-24°00’  24°43’-27°03’  38°50’-41°25’  39°01’-40°17’  40°44’-40°45’  32°32’-32°40’  37°00’-44°00’  22°53’-23°53’  35°00’-41°01’  27°02’-33°11’  35°00’-35°05’  29°00’-32°00’  27°00’-32°07’  23°00’-32°00’  17°17’-40°50’  46°50’-46°50’  41°50’-51°30’  47°42’-48°02’  47°17’-47°23’ | 4000-4650  1400-1700  0-2000  1000-2100  3050-4500  2100-3000  0-3000  0-500  1500-3000  1000-2300  1800-1800  3400-3750  0-2100  1500-2000  1700-2100  2200-3900  2436-2460  1520-1950  1520-1700  0-250  700-3000  2400-3200  1000-2100  400-520  500-1000  754-954  2850-3672  950-2000  0-1800  2100-3000  3200-3500  2600-2800  1500-2500  3300-3600  2200-2200  0-250  1250-3370  0-2500  3000-4700  2200-2300  600-1400  0-1600  0-1050  400-800  3680-4400  2820-3300  2500-3000  1800-2590  140-150  2438-4080  0-1800  2400-2400  1800-2800  0-750  3048-4755  0-800  610-1600  800-1000  800-1100  1100-1252  0-1000  85-250  850-920  1300-1400  280-350  700-1000  800-1400  195-1100  4100-4900  2500-3500  4300-4570  2300-2450  3700-4200  3200-3600  3500-3900  3500-4600  3600-4200  3200-5100  4000-4600  2850-3500  4240-4400  2400-2500  2400-3700  1960-4200  2370-3000  4000-4500  3700-4300  2050-2200  1200-1900  3060-4020  0-2000  900-900  690-820  400-2000  800-3000  1000-1000  4650-4650  0-1100  1428-1521  3060-5000  800-2450  800-1100  914-1041  610-1029  900-2070  740-1023  900-1770  3500-4800  1600-3000  500-1903  931-1355  990-1014  1830-2200  0-100  50-1200  0-1000  0-250  1800-1800  500-1000  0-2050  1000-2100  0-2600  1000-1020  780-1500  1000-1200  1500-1600 |
